# A Case of Lacrimal Sac Sarcoma

**DOI:** 10.7759/cureus.79121

**Published:** 2025-02-16

**Authors:** Yamamoto Yuki, Kenta Uemura, Yuichi Teranishi, Masaya Oishi, Kishiko Sunami

**Affiliations:** 1 Department of Otorhinolaryngology, Osaka Metropolitan University Graduate School of Medicine, Osaka, JPN

**Keywords:** chronic dacryocystitis, lacrimal sac sarcoma, lacrimal sac tumor, nasal endoscopy, tumor resection

## Abstract

Malignant tumors arising from the lacrimal sac are rare and present with diverse clinical symptoms. Many primary malignant tumors of the lacrimal sac exhibit lacrimation and are easily misdiagnosed as chronic dacryocystitis, delaying diagnosis and treatment. In this report, we describe a case of a sarcoma that was thought to have originated in the lacrimal sac.

## Introduction

Although primary tumors of the lacrimal sac are relatively rare, malignant tumors are common, accounting for approximately 55% of cases [1]. Among malignant tumors, squamous cell carcinoma is the most common followed by basal cell carcinoma, adenocarcinoma, mucoepidermoid carcinoma, adenoid cystic carcinoma, transitional epithelial carcinoma, malignant melanoma, and malignant lymphoma. Moreover, the incidence of sarcoma is rare. Prognosis is related to pathological type and staging, with early diagnosis and treatment being particularly important. The efficacy of nonsurgical resection, radiotherapy, and chemotherapy is not clear, and there are currently no uniform standards for the treatment of lacrimal sac malignancies [2-7]. In Japan, however, there have been no reports. In this report, we describe a case of a sarcoma thought to have originated in the lacrimal sac.

## Case presentation

We report the case of a 73-year-old woman whose chief complaint was bloody rhinorrhea. Her medical history includes uterine myoma, left trigeminal neuralgia, hypertension, and dyslipidemia. She has a drinking history of consuming 350 ml of beer daily for 60 years (from age 20 to present) and a smoking history of 20 cigarettes per day for 30 years (from age 20 to 50).

**Figure 1:**
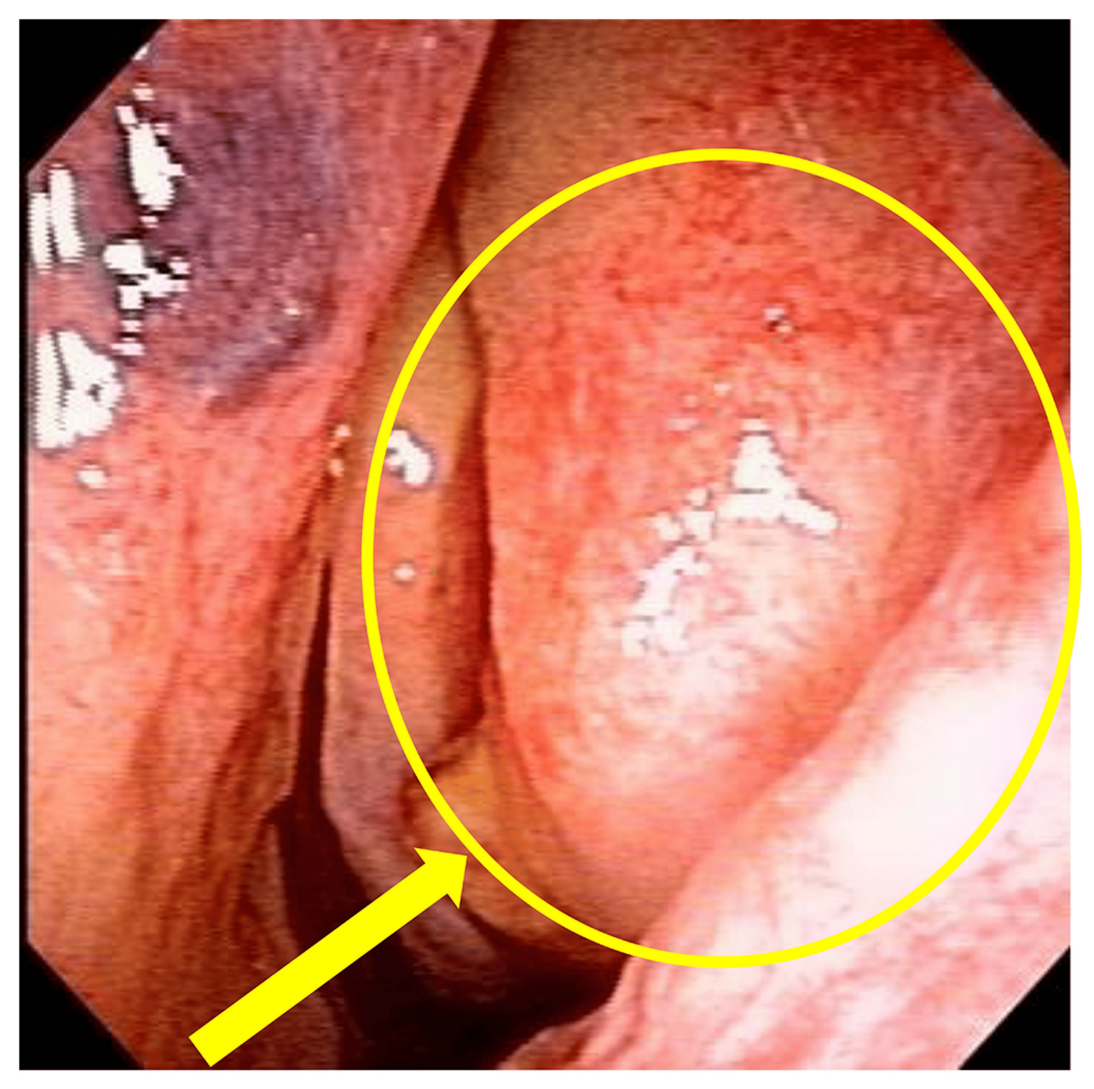
Status at the time of initial examination Source: Image of the patient showing the condition/feature. Informed consent for the use of this image was obtained from the patient.

In August 2017, the patient visited her local otorhinolaryngologist due to her complaint of bloody rhinorrhea. A plain paranasal sinus X-ray revealed a left-sided predominant sinus shadow, which was diagnosed as chronic sinusitis and treated conservatively; however, there was no improvement. Consequently, the patient was referred to our department in November 2017 for further examination and treatment. During the initial examination, it was noted that the ethmoid uncinate process was protruding anteriorly and viscous nasal discharge was observed posteriorly (Figure 1).

**Figure 2:**
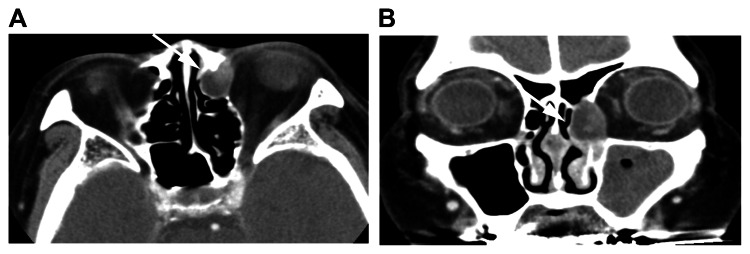
Paranasal sinus CT scan: (A) axial image and (B) coronal image A neoplastic lesion of 12×18×20 mm in size with clear margins and partial contrast enhancement is seen protruding from the lacrimal sac into the anterior ethmoid sinus. CT: computed tomography Source: Image of the patient showing the condition/feature. Informed consent for the use of this image was obtained from the patient.

A paranasal sinus computed tomography (CT) scan revealed a neoplastic lesion measuring 12×18×20 mm with clear margins and partial contrast enhancement, protruding from the lacrimal sac into the anterior ethmoid sinus (Figure 2).

**Figure 3:**
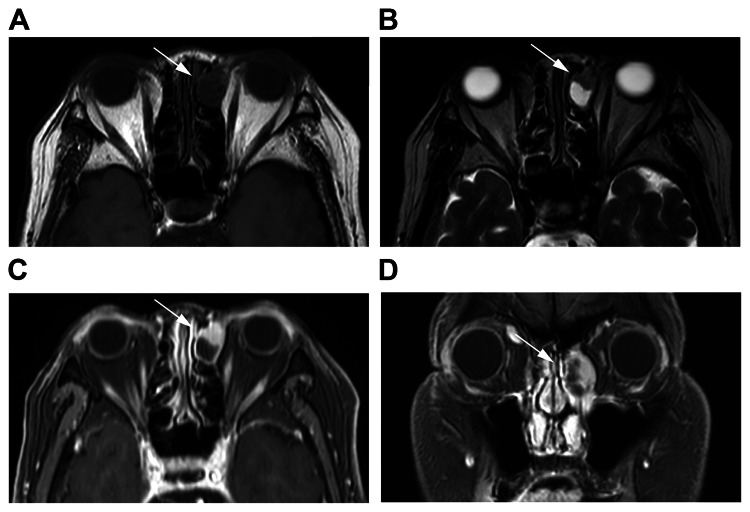
MRI scan of the paranasal sinuses: (A, B) axial T1-weighted images and (C, D) coronal T2-weighted images A lesion with a nearly isointense signal compared to muscle on T1-weighted images and a mixture of hypo- and hyperintense regions on T2-weighted images is seen. MRI: magnetic resonance imaging Source: Image of the patient showing the condition/feature. Informed consent for the use of this image was obtained from the patient.

Additionally, a magnetic resonance imaging (MRI) scan of the paranasal sinuses showed a lesion with a nearly isointense signal compared to muscle on T1-weighted images and a mixture of hypo- and hyperintense regions on T2-weighted images (Figure 3).

**Figure 4:**
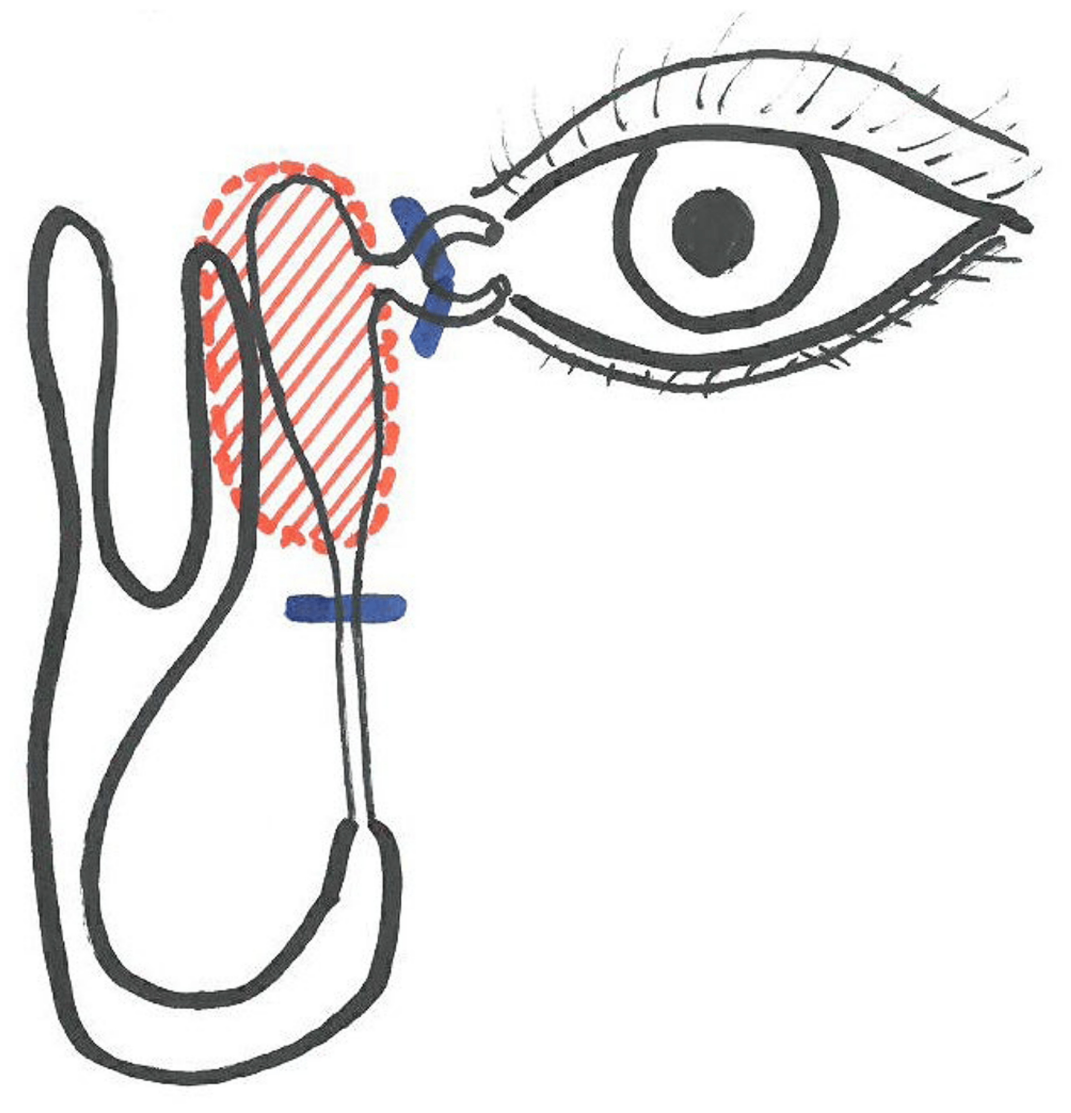
Surgical findings Source: Author's own work

The preoperative diagnosis was a lacrimal sac tumor. Furthermore, a tumor was identified through nasal endoscopy followed by left-sided excision of the uncinate process. Subsequently, the mucosa of the lacrimal sac area lateral to the nasal ridge was removed, and the bone was cut open with a drill to be able to check up to the frontal sinus (Draf II approach). The nasolacrimal duct was exposed inferiorly from the lacrimal sac; however, the tumor had spread posteriorly from the lacrimal sac. Although the nasolacrimal duct could be freed inferiorly, the lacrimal sac and tumor were difficult to detach, which suggested a primary tumor of the lacrimal sac. A specimen of the tumor was submitted to intraoperative rapid pathology, and based on the results, the tumor was diagnosed as malignant. Lateral dissection or resection was deemed difficult to perform endoscopically; thus, a concurrent external incision was used. A longitudinal incision was made medial to the left internal canthus, and the subcutaneous tissue was dissected. The medial palpebral ligament was identified and severed, and the tumor was identified deep inside the lacrimal sac. Lacrimal canaliculi from the upper and lower lacrimal points were confirmed with a lacrimal duct bougie and sectioned leaving a safety margin from the tumor. The tumor was dissected subperiosteally and dropped into the nose along with the medial wall of the orbit, after which the en bloc was removed through the anterior nares. Additionally, the orbital periosteum was removed in combination, and reconstruction was performed with a Super Fixove MX mesh plate (Teijin Medical Technologies, Osaka, Japan). Subsequently, the upper and lower lacrimal points were dilated, and a lacrimal fluid and tear duct silicone tube was placed (Figure 4).

**Figure 5:**
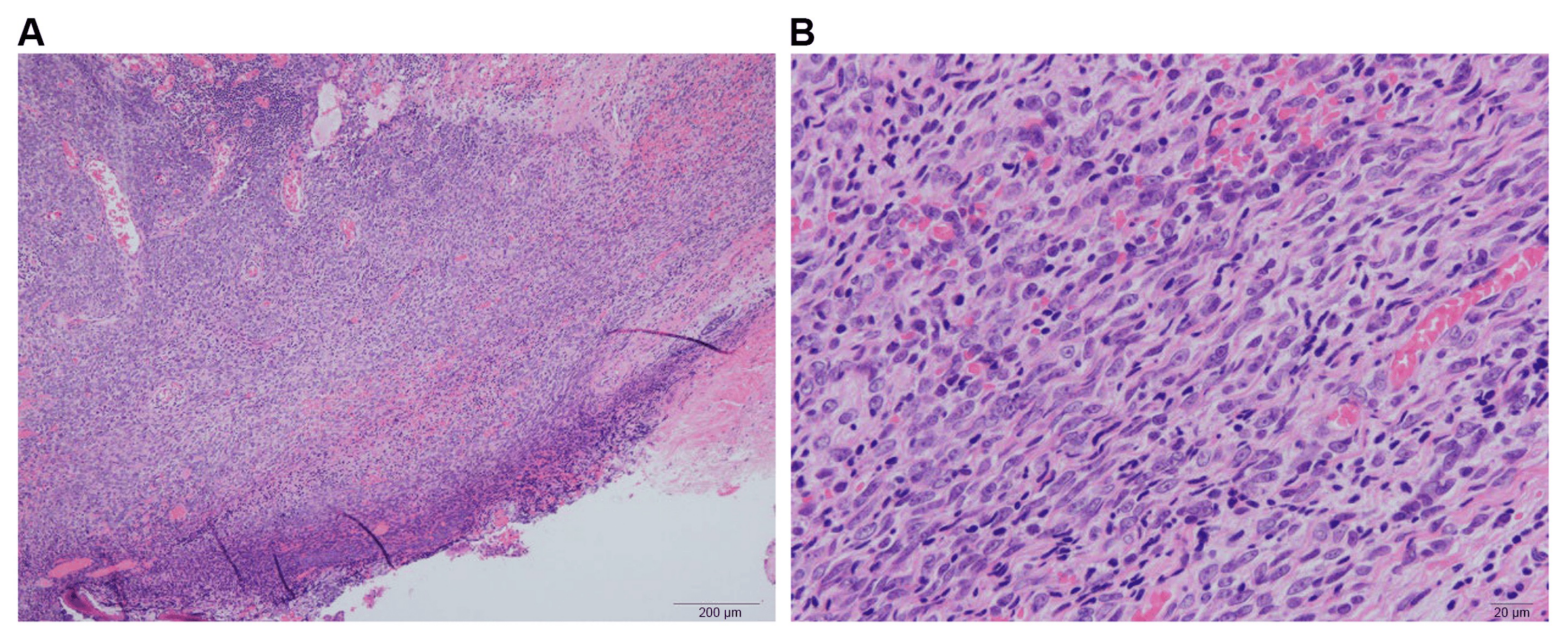
Histopathology results: (A) HE stain ×40 and (B) HE stain ×200 An unclassified spindle cell sarcoma, Grade 1, is indicated. HE: hematoxylin and eosin Source: Image of the patient showing the condition/feature. Informed consent for the use of this image was obtained from the patient.

Histopathology results indicated an unclassified spindle cell sarcoma, Grade 1 (Figure 5).

## Discussion

**Figure 6:**
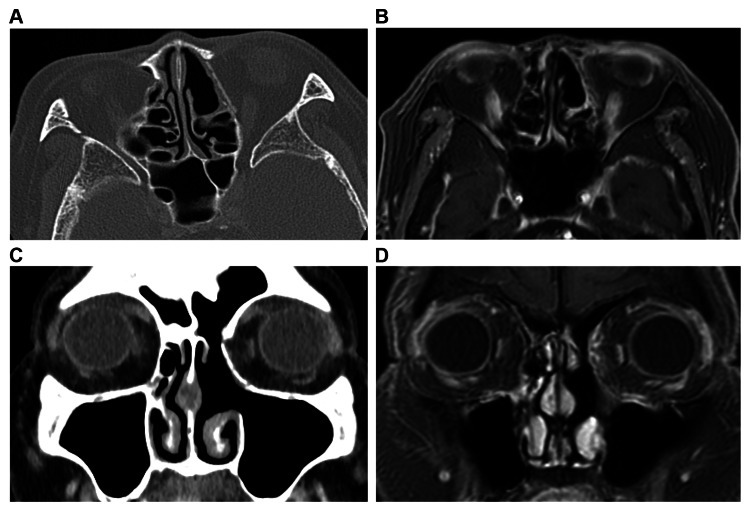
Postoperative CT/MRI scan: (A) CT axial image, (B) CT coronal image, (C) MRI axial T1-weighted image, and (D) MRI coronal T1-weighted image No evidence of postoperative recurrence is seen. CT: computed tomography; MRI: magnetic resonance imaging Source: Image of the patient showing the condition/feature. Informed consent for the use of this image was obtained from the patient.

Lacrimal sac tumors can be divided into epithelial and non-epithelial neoplasms. Benign epithelial tumors include squamous and transitional epithelial papillomas, oncocytomas, and benign mixed tumors. Malignant neoplasms include squamous cell carcinoma, transitional epithelial carcinoma, adenocarcinoma, mucoepidermoid carcinoma, and adenoid cystic carcinoma, as well as poorly differentiated cancers. Non-epithelial tumors include fibrous histiomas, lymphoid lesions, malignant melanoma, hemangiopericytoma, lipoma, granulocytic sarcoma, and neurofibromas. Approximately half of the tumors originating from the lacrimal sac are malignant. Particularly, invasive transitional epithelial carcinoma often presents with local recurrence and can metastasize, becoming fatal [1]. Malignant tumors of the lacrimal sac are rare and commonly occur in people aged 50-60 years, without gender- and racial-specific cancer risk. Malignant tumors of the lacrimal sac are characterized by slow onset, and because there are no specific clinical symptoms, they are generally misdiagnosed. Additionally, the time from illness onset to the time of the initial patient visit is usually long [2-6]. Malignant tumors arising in the lacrimal sac present various clinical symptoms, such as lacrimation, pyorrhea, local mass in the lacrimal sac, invasion into the nasal cavity and orbit, and local skin rupture due to tumor growth in the early stages, with regional lymphadenopathy and generalized metastases in advanced stages. The most common clinical symptoms of malignancy arising in the lacrimal sac are swelling of the lacrimal sac area and lacrimation, which are similar to those observed in chronic dacryocystitis. Therefore, this cancer is easily misdiagnosed as chronic dacryocystitis. Rare symptoms include hemolacria with redness and swelling of the lacrimal sac area, and attention should be paid to these symptoms. The prognosis of lacrimal sac tumors is related to the pathological type and staging, with early diagnosis and treatment being particularly important. Currently, in the treatment of lacrimal sac malignancies, with the exception of surgical resection, the therapeutic efficacy of radiation therapy and chemotherapy is not clear [1]. In the case of malignant tumors confined to the lacrimal sac, surgery is performed to remove the mass, nasolacrimal duct, and surrounding periosteum. For most high-grade tumors, the extent of resection should include the nasal bones and the orbital bone. For most malignant tumors that have invaded surrounding tissues, the tumor is excised as thoroughly as possible to remove the tumor margins. Postoperative radiation therapy improves local control rates and decreases tumor recurrence as well as metastasis rates [6,7]. In this case, there were no symptoms, such as dacryocyst swelling or lacrimation, and no findings suggestive of malignancy, such as bone destruction, on imaging studies. Therefore, it was difficult to assume malignancy preoperatively. However, the diagnosis of malignancy was made based on intraoperative findings and rapid pathology. The present case had a malignant tumor; therefore, surgical resection was performed with concurrent resection of the orbital periosteum to ensure an adequate safety margin. Because the resection margins were negative, we decided to monitor the patient's progress without performing additional treatment. Currently, seven years have passed since the surgery, and the patient has progressed without recurrence (Figure 6). We confirm that prior written informed consent was obtained from the patient for both treatment and publication of this case.

## Conclusions

Primary malignant tumors of the lacrimal sac are rare in clinical practice; therefore, more cases need to be reported, and experiences in diagnosis and treatment should be compiled to improve the treatment outcomes and management of this cancer. Symptoms of lacrimation and imaging studies with nasolacrimal duct dilation and bone destruction should be noted as a possible lacrimal sac malignancy.
